# A Systematic Review Investigating Maternal Nutrition During Pregnancy After Bariatric Surgery

**DOI:** 10.1007/s11695-023-06565-8

**Published:** 2023-04-22

**Authors:** Taylor M. Guthrie, Clare F. Dix, Helen Truby, Sailesh Kumar, Susan J. de Jersey

**Affiliations:** 1 Faculty of Medicine, Centre for Health Services Research, The University of Queensland, Herston, Brisbane, Queensland 4029 Australia; 2 Dietetics and Foodservices, Royal Brisbane Women’s Hospital, Metro North Health, Herston, Brisbane, Queensland 4029 Australia; 3 School of Human Movement and Nutrition Sciences, The University of Queensland, St Lucia, Brisbane, Queensland 4067 Australia; 4 Mater Research Institute and Faculty of Medicine, The University of Queensland, South Brisbane, Brisbane, 4101 Australia

**Keywords:** Pregnancy, Bariatric surgery, Nutrition, Diet, Micronutrients, Gestational weight gain, Perinatal outcomes

## Abstract

The aim of this review was to report on maternal diet, micronutrient supplementation, and gestational weight gain (GWG) during pregnancy following bariatric surgery and explore the impact on maternal micronutrient deficiency, offspring growth, and perinatal outcomes. A search in PubMed, CINAHL, EMBASE, and ProQuest in July 2022 returned 23 eligible studies (*n* = 30–20, 213). Diet was reported in two studies, supplementation in six and GWG in 19 studies. Although many women did not achieve healthy GWG, no consistent link with adverse outcomes was reported. Studies were grades II and III on the National Health and Medical Research Council evidence hierarchy and received a neutral or negative score on the Academy of Nutrition and Dietetics Quality Criteria Checklist, suggesting that methodological limitations impact the reliability of reported findings.

**Supplementary Information:**

The online version contains supplementary material available at 10.1007/s11695-023-06565-8.

## Introduction

It is now clear that bariatric surgery offers significant health benefits as a treatment for severe obesity [1, 2]. In pregnant women, however, the evidence for improved pregnancy and perinatal outcomes is mixed [3]. In many countries, women of childbearing age represent the majority of bariatric surgery recipients [4, 5]. By altering the shape, length, and physiology of the gastrointestinal tract, bariatric surgery results in a significant energy (calorie) deficit and promotes substantial weight loss [6]. However, post-surgery, many recipients continue to have a poor quality diet [7] and may experience nausea, vomiting, reflux, or dumping syndrome, all of which not only exacerbates nutritional intake [6] but also results in vitamin and mineral deficiencies [8, 9].

Maternal dietary intake is crucial to the wellbeing of the mother and fetus. Micronutrient deficiencies are associated with various maternal and perinatal complications [10]. Iron, vitamins A, B12, D, K, and folate deficiency have been reported during pregnancy following bariatric surgery [9, 11, 12]. Although prophylactic multivitamin-multimineral supplementation is recommended [9, 11, 12], there is limited evidence underpinning the doses required to prevent deficiency during pregnancy. Dietary energy intake is a key determinant of gestational weight gain (GWG) [10]. Both insufficient and excessive GWG, as defined by the Institute of Medicine recommendations according to pre-pregnancy body mass index (BMI), are correlated with adverse pregnancy and birth outcomes [13]. There is evidence that bariatric surgery reduces the risk of developing gestational diabetes mellitus (GDM) [14–17], pregnancy-induced hypertension [14, 16, 18, 19], pre-eclampsia [3, 14, 15, 17, 18, 20], and large for gestational age (LGA) infants [3, 14–20]. Conversely, studies suggest that bariatric surgery may increase the risk of small for gestational age (SGA) infants [3, 15, 18–20], and preterm birth [3, 15, 18–20]. These inconsistent findings point to the need for careful evaluation of available evidence regarding maternal nutrition and pregnancy outcomes post weight loss surgery. The aim of this systematic review thus was to synthesise the available literature relating to maternal diet, micronutrient supplementation, and GWG during pregnancy following bariatric surgery. A secondary aim was to explore the potential contribution of these inter-related factors on maternal micronutrient deficiency, offspring growth, and perinatal outcomes.

## Materials and Methods

A review protocol was developed (PROSPERO registration #CRD42022308295) according to the preferred reporting items for systematic review and meta-analysis protocols [21].

## Search Methods and Eligibility Criteria

Comprehensive searches (Supplement 1) including controlled vocabulary were used to search for studies published between 1980 and July 28, 2022 in PubMed, CINAHL, EMBASE, and ProQuest (grey literature). Studies were eligible for inclusion if they investigated any measure of dietary intake, micronutrient supplementation (including dose and adherence to supplementation regimen) or GWG in relation to maternal micronutrient deficiency, GDM, hypertension, pre-eclampsia, preterm birth, SGA or low birth weight, LGA or macrosomia. Included bariatric surgical procedures were adjustable gastric band (AGB), sleeve gastrectomy (SG), Roux-en-Y gastric bypass (RYGB), and one anastomosis gastric bypass (OAGB). Primary studies of any design were eligible for inclusion but excluded case studies, case series, conference abstracts, and reviews.

## Study Selection and Data Extraction

Article screening was conducted in Covidence™ which removed duplicates. Two authors (TG and CD), blinded to each other’s responses completed abstract and title screening. A third author (SdJ) resolved disagreements. The reference lists of the included studies were also searched for relevant articles. Data was extracted using the strengthening the reporting of observational in epidemiology nutrition and dietetics [22] and molecular epidemiology [23] frameworks. This was undertaken by two authors (TG and CD) and a maternal and fetal medicine specialist (SK) who reviewed data extraction for 10% of papers to ensure appropriate interpretation of clinical data.

The primary outcome of interest was maternal micronutrient deficiencies whilst secondary outcomes were GDM, pregnancy-induced hypertension, pre-eclampsia, preterm birth, small neonates (SGA or low birth weight), large neonates (LGA or macrosomia), and perinatal mortality (stillbirth or neonatal death after 20 weeks gestation). Additional data extracted to assist with interpretation and consideration of confounders, included: surgical procedure, surgery-to-conception interval, pre-pregnancy BMI, consideration of confounders to low serum micronutrient levels (gestation and inflammation), use of assisted reproductive technology, maternal demographics, smoking status, pre-existing diabetes, gestation at birth, and neonatal sex.

## Critical Appraisal

Two authors (TG and CD) established the risk of bias for each study using the Academy of Nutrition and Dietetics Quality Criteria Checklist [24]. This tool provides an overall rating of negative, neutral, or positive using 10 questions addressing bias in study recruitment, performance, detection, and attrition. The National Health and Medical Research Council (NHMRC) evidence hierarchy was applied to determine the level of evidence for individual studies, with reference to the type of research question and risk of bias [25].

## Results

**Fig. 1:**
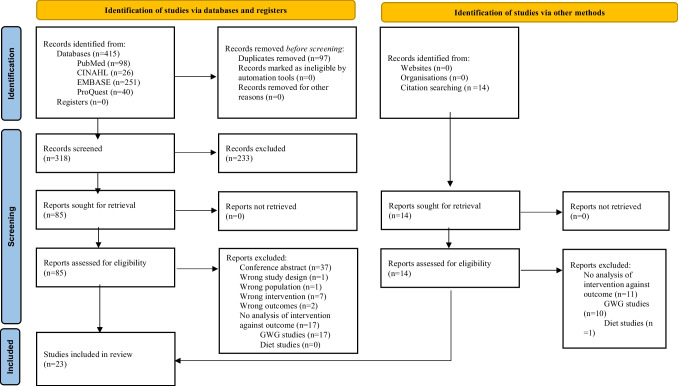
Preferred Reporting Items for Systematic Reviews and Meta-Analyses Diagram

The search returned 415 articles. After excluding duplicates (*n* = 97) and irrelevant studies (*n* = 233), 85 articles underwent full text screening (Fig. 1). After full text screening, 65 articles were excluded. An additional three articles were identified following review of reference lists. In total 23 papers met all eligibility criteria [26–48] and were included in the final analysis. Sample sizes ranged from 30 to 20,213 with a total of 4343 women post-bariatric surgery and 21,193 controls (the number of overlapping participants between four studies conducted in Brazil [30–32, 44] and two in Belgium [26, 27] was unclear). Study characteristics are summarised in Supplement 2.

## Critical Appraisal

Seventeen studies received a neutral rating and six received a negative rating (Table 1). Gaps existed in the description of participant characteristics, such as parity, smoking status, and ethnicity (checklist item two). Reporting of co-interventions and exposure to an intervention was limited, largely because all studies investigating micronutrient deficiency neglected to report on dietary intake or adherence with micronutrient supplementation (checklist item six). Both studies reporting dietary intake [29, 34] omitted relevant quality control information such as how portion sizes were estimated, the validation method applied to ensure accuracy of self-reported dietary intake, and the data source and method for nutrient calculations. Additionally, 61% (14/23) of studies did not use valid or reliable outcome measures (checklist item seven), due to omitting diagnostic criteria for GDM, micronutrient deficiency, or failure to consider relevant confounders to micronutrient levels. Limited description of statistical methods made it difficult to determine whether the analyses were appropriate, and 61% (14/23) of studies did not adjust for differences between comparison groups (checklist item eight). Baseline differences in maternal age, [33] use of assisted reproduction, [26] and pre-pregnancy BMI [36, 39, 40] may have impacted the findings of primary studies. Studies were grades II, III-2, and III-3 on the NHMRC evidence hierarchy. These variations in reporting precluded the application of meta-analysis.

**Table 1:**
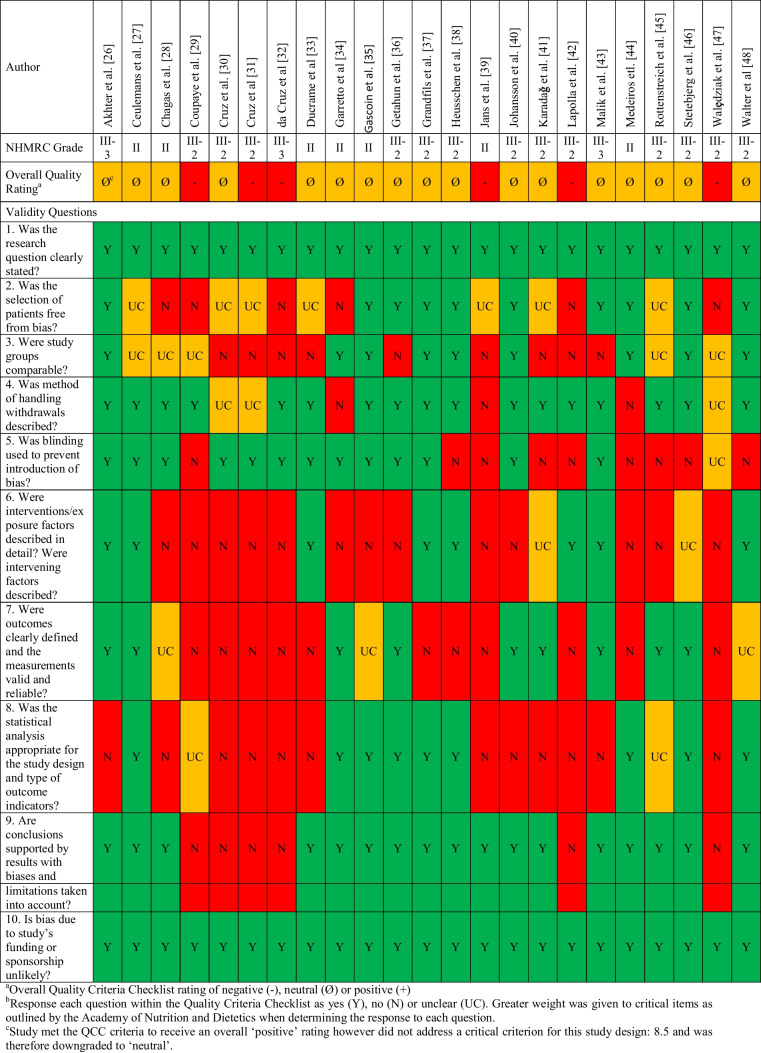
Critical appraisal of included studies using the quality criteria checklist and NHMRC evidence hierarchy

## Micronutrient Supplementation and Dietary Intake

Eight studies [29–34, 39, 44] investigated micronutrient intake, only two [29, 34] measured dietary intake (Supplement 3). Studies included women who had undergone malabsorptive procedures exclusively [28, 30–32, 44] or combined data from recipients of restrictive and malabsorptive procedures [29, 34, 39]. In a study of 123 women post-SG and RYGB, Coupaye et al. [29] examined iron intake from diet and supplements in relation to the neonate’s birthweight (z-score). Transferrin saturation was the only independent factor found to influence birthweight. A retrospective cohort study [34] investigated vitamin A deficiency (using serum β-carotene) in post-operative women compared to unmatched pregnant women, and reported no difference in the incidence of deficiency between groups. Women post-bariatric surgery did have lower serum β-carotene, however, this was not significant after adjusting for β-carotene intake measured via a food frequency questionnaire [34].

Six studies reported micronutrient supplementation during pregnancy. Five [28, 30–32, 44] included only women who took at least 80% of prescribed supplements, though four of these appeared to have an overlapping study population [30–32, 44]. Amongst women taking 5000 IU/d of vitamin A [28, 31, 32], 57 to 75% had retinol deficiency, and 66 to 90% β-carotene deficiency. Two studies [30, 44] of women taking 600 IU/d vitamin D post-RYGB found varied prevalence of deficiency (first trimester: 30 to 83%, second trimester: 20 to 87%, third trimester: 39 to 90%). The threshold for deficiency for one study [30] was unclear and may account for these differences. Jans et al. [39] reported participants’ refusal to take micronutrient supplements, but did not explain how this was determined. Two studies [30, 32], with an overlapping study population, reported deficiency rates according to the surgery-to-conception interval, but did not detect a difference between groups. There were no studies examining the impact of dietary intake or micronutrient supplementation on hypertension, pre-eclampsia, GDM, or neonatal mortality.

## Gestational Weight Gain

Nineteen studies reported GWG. Three [41, 42, 45] exclusively investigated women after restrictive bariatric surgery procedures, six malabsorptive procedures [30, 31, 35, 44, 48, 49] and 10 combined data from a mix of surgery types [26, 27, 29, 33, 36–38, 40, 43, 47].

Adherence to the Institute of Medicine [13] GWG guidelines was reported by 12 studies [26–28, 31, 36–38, 44–46, 48, 50]. Between 17 and 67% of women achieved the recommended GWG, 18 to 73% of women had inadequate GWG, and 7 to 48% of women had excessive GWG (Table 2). Akhter et al. [26] conducted a case-control study comparing GWG in pregnancies resulting in SGA offspring (cases) and appropriate weight for gestational age neonates (controls) in women post-bariatric surgery. No difference in adherence to GWG recommendations were identified, although women who gave birth to SGA neonates had lower mean weight gain (9.8 kg vs 13.0 kg, *p* = 0.029). In a study comparing women post-bariatric surgery to women with a pre-pregnancy BMI >35kg/m^2^ without bariatric surgery [36], the authors also reported no difference in adherence to GWG recommendations but higher mean weight gain in post-operative women (8.9 kg vs 4.2 kg, *p* < 0.01). Studies investigating the influence of surgery-to-conception interval on maternal attainment of GWG recommendations compared different intervals, making it difficult to synthesise their findings [38, 45, 46].

**Table 2:** Alignment with the Institute of Medicine gestational weight gain recommendations

Reference	Sample size (*n* =)	Inadequate GWG	Adequate GWG	Excessive GWG
Studies including recipients of restrictive bariatric surgery procedures (AGB, SG)				
Rottenstreich et al. [45]	*n* = 196	< 6 months post-surgery 73.9% ≥ 6 months post-surgery 18.5%	< 6 months post-surgery 17.4% ≥ 6 months post-surgery 38.7%	< 6 months post-surgery 8.7% ≥ 6 months post-surgery 42.8% *p* < 0.001
Studies including recipients of malabsorptive bariatric surgery procedures (RYGB, OAGB)				
Chagas et al. [28]	*n* = 30	50%	37%	13%
Cruz et al. [31]	*n* = 30	51.7%	34.5%	13.8%
Medeiros et al. [44]	*n* = 46	48.9%	44.4%	6.7%
Stenetebjerg et al. [46]	*n* = 71	Overall 42.6% < 18 months post-surgery 51.5% > 18 months post-surgery 28.6%	Overall 24.1% < 18 months post-surgery 24.2% > 18 months post-surgery 23.8%	Overall 33.3% < 18 months post-surgery 24.2% > 18 months post-surgery 47.6%
Walter et al. [48]	*n* = 132	31%	33.6%	35.3%
Studies combining data from recipients of restrictive and malabsorptive bariatric surgery procedures				
Akhter et al. [26]	*n* = 122 (*n* = 25 cases)	Cases 44% Controls 16.5%	Cases 28% Controls 26.8%	Cases 28% Controls 51.5%
Ceulemans et al. [27]	*n* = 127	24%	20%	56%
Ducrame et al. [33]	*n* = 87	Not reported	SG 60% RYGB 67.6%	Not reported
Getahun et al. [36]	*n* = 20,213 (*n* = 1886 cases)	Cases 30.2% Controls 45.1%	Cases 20% Controls 20.5%	Cases 48.0% Controls 33.3%
Grandfills et al. [37]	*n* = 337	35%	26.7%	38.3%
Heusschen et al. [38]	*n* = 196	Overall 40.6% ≤ 12 months post-surgery 75% 12 to 24 months post-surgery 24.4% > 24 months post-surgery 32.6% *p* < 0.001	Overall 29.4% ≤ 12 months post-surgery 20% 12 to 24 months post-surgery 41.5% > 24 months post-surgery 28.1%	Overall 30% ≤12 months post-surgery 5% 12 to 24 months post-surgery 34.1% >24 months post-surgery 39.3% p0.002–0.004

*AGB* adjustable gastric band, *SG* sleeve gastrectomy, *OAGB* one anastomosis gastric bypass, *RYGB* Roux-en-Y gastric bypass, *GWG* gestational weight gain

Six studies reported on adherence to GWG recommendations and incidence of GDM, hypertension, pre-eclampsia, or neonatal mortality, and found no associations [27, 36–38, 46, 48]. Three studies reported an increased risk of preterm birth amongst women with inadequate GWG [27, 37, 38], whereas two did not [46, 48]. Similarly, Ceulemans et al. [27] and Walter et al. [48] found inadequate GWG increased the risk of SGA neonates, whereas four studies did not [26, 37, 38, 46]. Seven studies analysed GWG as a continuous variable [29, 33, 36, 40, 41, 45, 47]; however, only three of these studies considered pre-pregnancy BMI in their analysis [29, 36, 41]. Coupaye et al. [29] reported a statistically significant relationship between total GWG and birth weight z-score, but did not analyse the impact on risk of SGA neonates. Karadaǧ et al. [41] reported that total GWG was not a predictor of SGA neonates. Johansson et al. [40] (*n* = 2952) and Getahun et al. [36] (*n* = 20,213) conducted large cohort studies comparing pregnancy outcomes post-bariatric surgery with controls. Both studies reported that adjusting for total GWG did not change odds ratios for GDM, preterm birth, SGA, LGA, or neonatal mortality.

## Discussion

This systematic review identified no clear and consistent relationships between dietary intake, supplementation, or GWG with micronutrient deficiency. Maternal and neonatal outcomes were largely investigated in studies examining GWG. Some studies found a relationship between GWG and the risk of preterm birth [27, 37, 38] and SGA neonates [27, 47], but not with other adverse outcomes. Half of the included studies (12/23) combined data from recipients of restrictive and malabsorptive procedures [26, 27, 29, 33, 34, 36–40, 43, 47]. Studies reporting solely on restrictive bariatric surgery procedures were scarce, limiting generalisability to the 40 to 70% of women who have undergone AGB or SG [5, 51].

Due to the risk of micronutrient deficiency following bariatric surgery, guidelines recommend pregnant women supplement their diet with multivitamins containing folic acid, thiamine, iron, copper, zinc, selenium, alongside vitamins A, D, E, and K [9, 11, 12]. Consistent with prior reviews [52, 53], studies in this review reported high rates of micronutrient deficient women despite their adherence to micronutrient supplementation. Most studies (5/8) excluded women with poor adherence to micronutrient supplementation, so adherence to recommended supplementation amongst pregnant women with a history of bariatric surgery is unclear. Although seldom investigated by studies in this review, the link between micronutrient deficiency and pregnancy complications is well established in the broader obstetric population. These include: congenital anomalies (folate [54]), GDM (vitamin D [55]), hypertension and pre-eclampsia (zinc [56], calcium and vitamin D [55]), preterm birth (iron [57], vitamin B12 [58]), and SGA (vitamin B12 [58], iron [57] and zinc [56]). Studies did not establish a relationship between micronutrient supplementation and deficiency risk, which may relate to confounders like dietary intake. For example, despite similar supplement regimens and adherence, women post-RYGB in Chagas et al.’s [28] study had approximately twice the rate of vitamin A deficiency compared to Coupaye et al.’s [29] (63% vs 31% in the second trimester). It is not possible to ascertain whether this difference is due to dietary behaviours, or other confounders. Further research examining dietary intake alongside micronutrient supplementation is required to unravel the relationship between nutrient consumption and micronutrient deficiency.

Current clinical practice guidelines offer little direction to clinicians who provide dietary advice to pregnant women. Most guidelines recommend that dietary advice is individualised to maternal characteristics and GWG [9, 11, 12]. It has been suggested that a minimum of 60 g protein intake is appropriate during pregnancy post-bariatric surgery [9]. However, this neglects the theoretical increases in protein requirements required to achieve a healthy GWG [59]. Only one paper in this review reported protein intake but did not explore its impact on GWG [29]. This is a significant gap as studies suggest a large proportion of women are not achieving healthy GWG [26–28, 31, 33, 36–38, 44, 46, 48]. The importance of healthy GWG for optimising maternal and offspring health as well as life-long risks of obesity and chronic disease has been established in women who have not had bariatric procedures [60]. Inadequate GWG has been associated with increased risk of preterm birth, and SGA in the broader obstetric population [13]. This was echoed in some of the studies included in this review [27, 37, 38, 47]. Although not reported in women post-bariatric surgery, excess GWG has been linked to GDM, hypertension, and LGA infants [32]. Larger studies with methodological improvements may provide a clearer understanding of optimal GWG for women post-bariatric surgery.

The strengths and weaknesses of this review require consideration. This review followed a pre-determined protocol that specified inclusion/exclusion criteria and data extraction processes. Although language filters were not used, the reliance on English-language databases may have limited the results. To identify all relevant research, a grey literature database was used and reference lists were searched for relevant articles. The exclusion of studies that did not report adherence to micronutrient supplementation reduced the number of studies available for inclusion. However, as supplement adherence has been reported as low as 33% 5 years post-operatively [61], this information is crucial to understanding the impact of micronutrient supplementation. This review was strengthened by performing article screening in duplicate, with a third author to resolve conflicts and performing data extraction in duplicate, using a third author with specialist clinical knowledge to ensure appropriate interpretation.

Limitations to the included studies have implications for the interpretation of our findings. Common risk factors for adverse pregnancy outcomes such as smoking were often unaccounted for. Smoking, for example, approximately doubles the risk of preterm birth [62], but was only reported by 10 of the 23 included studies. Dietary intake studies also did not follow existing best practice recommendations for research [63]. Additionally, many studies examining GWG as a continuous variable did not adjust for pre-pregnancy BMI [33, 40, 45, 47]. A significant body of literature that suggests women with a higher pre-pregnancy BMI require less GWG to achieve optimal maternal and perinatal health outcomes [13]. Women with a higher BMI are at greater risk of excessive GWG as well as multiple complications [13], therefore, considering pre-pregnancy BMI when interpreting GWG data is imperative. Future research addressing these methodological limitations may enable meta-analysis and provide guidance for clinicians.

Our results indicate that despite adherence to micronutrient supplementation, high rates of micronutrient deficiency during pregnancy were reported in women post-bariatric surgery. Large proportions of women had GWG outside recommendations, though an associated link with adverse outcomes was not consistently identified. Most studies omitted critical information regarding methods of measuring dietary intake and baseline characteristics of participants which precluded comparison. This regrettably leads to an incomplete picture of maternal nutrition following bariatric surgery and challenges the ability to draw firm conclusions about which elements of nutrition post-bariatric surgery are critical to optimise pregnancy and perinatal outcomes.

## Supplementary information

ESM 1:
