# Supplementary material for: A Systematic Review Investigating Maternal Nutrition During Pregnancy After Bariatric Surgery
Source: Obes Surg. 2023 Apr 22;33(6):1857–65. doi: 10.1007/s11695-023-06565-8 (PMC10234856; doi:10.1007/s11695-023-06565-8)
Supplement: Supplementary file 1 — ESM 1: [file 11695_2023_6565_MOESM1_ESM.pdf]

Supplement 1

Search terms (not including controlled vocabulary)

Date range – 01/01/1980-28/07/2022

Filters applied: humans only

Search fields: title and abstract

((“Infant, Newborn” OR “Pregnancy” OR Pregnan# OR Gestation OR Matern* OR Offspring OR F$etal OR Neonat* OR F$etus OR Gravid) AND (“Bariatric Surgery” OR “Bariatric surger#” OR “Gastric band” OR “Gastric sleeve” OR Gastrectomy OR “Gastric bypass” OR “Roux-en-Y” OR “Metabolic surgery” OR “Weight loss surgery” OR “Stomach stapling”) AND (“Gestational weight gain” OR Macronutrient OR Protein OR Carbohydrate OR Fat OR Energy OR Kilojoule OR Calorie OR “Diet quality” OR “Oral intake” OR Diet* OR Micronutrient OR “Trace element” OR Vitamin OR Mineral OR OR Supplement* OR “Vitamin A” OR Retinol OR Carotene OR “Beta carotene” OR “Vitamin B1” OR Thiamin OR Thiamine OR “Vitamin B6” OR Pyroxidine OR “Vitamin B12” OR Cobalamin OR “Vitamin D” OR Calciferol OR Folate OR “Folic acid” OR Zinc OR Copper OR Iron OR Selenium OR “Vitamin K” OR Phylloquinone OR “Vitamin E” OR Alpha-tocopherol OR Iodine OR Magnesium OR Calcium OR Supplement* OR Multivitamin) AND (Deficiency OR Deficient OR Depletion OR Anaemi# OR Haemoglobin OR Malnutrition OR avitaminosis OR “Small-for-gestational-age” OR “Growth-restriction” OR “Low-birth-weight” OR Preterm OR Prematur# OR “gestational diabetes” OR “gestational diabetes mellitus” OR hypertension OR “pregnancy-induced hypertension” OR “large for gestational age” OR macrosomia OR pre-eclapmsia OR “perinatal-mortality”)) NOT (title only) “review”

Supplement 2: Included study characteristics

Reference, study design, country	Sample size (n=), study aim and inclusion criteria	Procedure, surgery-to-conception interval (months, mean(SD))	Maternal age, parity, pre-pregnancy BMI (mean(SD))
Studies including recipients of restrictive bariatric surgery procedures (AGB, SG)			
Karadağ et al. 2020 [41]<br>Retrospective cohort study<br>Turkey	n=144<br>Aim: Compare GWG and pregnancy outcomes in women falling pregnant ≤12 months and >12 months post-SG and with controls.<br>Cases (n=90): Pregnant women post SG known to single centre.<br>Controls (n=54): Pregnant women with pre-pregnancy BMI >30 kg/m^2 known to single centre.	All SG<br>Surgery-to-conception:<br>≤12 months 53%<br>>12 months 47%	Maternal age:<br>≤12 months post-surgery 30.3(5.1)<br>>12 months post-surgery 28.8(4.7)<br>Controls 27.5(3.9)<br>Parity (mean(range)):<br>≤12 months post-surgery 1(0 to 3)<br>>12 months post-surgery 1(0 to 2)<br>Controls: 1(0 to 2)<br>Pre-pregnancy BMI:<br>≤12 months post-surgery 32.8(3.6)<br>>12 months post-surgery 28.9(2.8)<br>Controls 31.1(3.1)
Lapolla et al. 2010 [42]<br>Retrospective cohort study<br>USA	n=230<br>Aim: Compare GWG and pregnancy outcomes post-AGB to controls.<br>Cases (n=83): Women with a pregnancy post-AGB.<br>Controls:<br>1) Cases with a pregnancy before AGB,<br>2) Obese controls: women without bariatric surgery with pre-pregnancy BMI >40 kg/m2 from an Italian database.<br>3) Healthy controls (n=858): women with healthy BMI used to compare select outcomes.	All AGB<br>Surgery-to-conception:<br>37.2(25.2)	Maternal age:<br>Cases 31.4(4.6)<br>Obese controls 33.0(5.1)<br>Healthy controls 32.3(4.2)<br>Parity: Not reported<br>Pre-pregnancy BMI:<br>Cases 35.0(7.3)<br>Obese controls 39.4(3.8)<br>Healthy controls 22.2(1.4)

Rottenstreich et al. 2021 [45]<br><br>Retrospective cohort study<br><br>Israel	n=196<br><br>Aim: Compare GWG and pregnancy outcomes in women who conceived <6 months and ≥6 months post-SG.<br><br>Inclusion: Pregnant women post-SG attending a single centre.	All SG<br><br>Surgery-to-conception (median (IQR)):<br>25(13 to 41)	Maternal age (median (IQR)):<br><6 months post-surgery 30(27 to 35)<br>≥6 months post-surgery 32(29 to 36)<br><br>Nulliparous:<br><6 months post-surgery 8.8%<br>≥6 months post-surgery 19.7%<br><br>Pre-pregnancy BMI (median (IQR)):<br><6 months post-surgery 34.2(31.2 to 39.4)<br>≥6 months post-surgery 29.4(27.1 to 32.0)
Studies including recipients of malabrosptive bariatric surgery procedures (RYGB, BPD)			
Chagas et al. 2016 [28]<br><br>Prospective cohort study<br><br>Brazil	n=30<br><br>Aim: Evaluate relationship between GWG, vitamin A and zinc status in pregnancy post-RYGB.<br><br>Inclusion: Women post RYGB attending a single centre with ≥80% adherence to prescribed supplements.	RYGB only<br><br>Surgery-to-conception:<br>17.7(9.1)	Maternal age: 30.2(4.4)<br><br>Parity: Not reported<br><br>Pre-pregnancy BMI: 27.4 (3.3)
Cruz et al. 2020[a] [30]<br><br>Retrospective cohort study<br><br>Brazil	n=119<br><br>Aim: Compare serum vitamin D, calcium and parathyroid hormone in women post-RYGB who fell pregnant to those who did not.<br><br>Cases (n=40): Women post-RYGB aged 21-39 with a singleton pregnancy, <13 weeks gestation with ≥80% adherence to prescribed supplements.<br><br>Controls (n=79): Non-pregnant women post RYGB matched for age and pre-operative BMI.	RYGB only<br><br>Surgery-to-conception:<br>≤12 months 25%<br>>12 months 75%	Maternal age:<br>Cases 31.9(3.9)<br>Controls 30.4(4.3)<br><br>Parity: Not reported<br><br>Pre-pregnancy BMI: Not reported

Cruz et al. 2017[a] [31]<br>Retrospective cohort study<br>Brazil	n=30<br>Aim: Compare serum vitamin A and GWG post-RYGB.<br>Inclusion: Pregnant women post RYGB aged 22 to 39 attending a single centre with ≥80% adherence to prescribed supplements.	RYGB only<br>Surgery-to-conception: 17.7(9.1)	Maternal age: 30.3(4.4)<br>Parity: Not reported<br>Pre-pregnancy BMI: 27.4(3.3)
da Cruz et al. 2018[a] [32]<br>Retrospective cohort study<br>Brazil	n= 116<br>Aim: Compare serum vitamin A in women post RYGB who fell pregnant to those who did not.<br>Cases (n=39): Pregnant women aged 20 to 39 ≤24 months post-RYGB with ≥80% adherence to prescribed supplements.<br>Controls (n=77): Non-pregnant women aged 20-39, ≤24 months post-RYGB.	RYGB only<br>Surgery-to-conception:<br><12 months 18%<br>12 to 24 months 82%	Maternal age:<br>Cases 32.7(4.9)<br>Controls 30.8(4.5)<br>Parity: Not reported<br>Pre-Pregnancy BMI: Not reported
Gascoin et al. 2017 [35]<br>Prospective cohort study<br>France	n=112<br>Aim: Compare maternal serum micronutrients and GWG in pregnant women post-RYGB to controls.<br>Cases (n=56): Pregnant women post RYGB known to a single centre.<br>Controls (n=56): Non-obese women birthing in the same period matched for age, parity and smoking status.	All RYGB<br>Surgery-to-conception: 32(14)	Maternal age:<br>Cases 30.5(4.2)<br>Controls 30.3(6.0)<br>Parity:<br>Cases 2.2(1.3)<br>Controls 2.2(1.1)<br>Pre-pregnancy BMI:<br>Cases 30.1(6.0)<br>Controls 22.3(4.0)
Medeiros et al. 2016[a] [44]<br>Prospective cohort study	n=46<br>Aim: Assess vitamin D status and GWG throughout pregnancy post-RYGB.	All RYGB<br>Surgery-to-conception: 23.7(20.7)	Maternal age: 30.6(4.6)<br>Parity: 0.9(0.8)<br>Pre-pregnancy BMI: 27.7(3.9)

Brazil	Inclusion: Women post-RYGB, aged ≥20, with a singleton pregnancy presenting for care before 11 weeks gestation.		
Stentebjerg et al. 2017 [46]<br>Retrospective cohort study<br>Denmark	n=76<br>Aim: Compare GWG and pregnancy outcomes in women pregnant <18 months and >18 months post-RYGB.<br>Inclusion: Women post-RYGB with a singleton pregnancy.<br>Controls: National data reported from Danish Health and Medicine Authority (n= not reported).	All RYGB<br>Surgery-to-conception (median (IQR)): 14(7 to 22)	Maternal age (median (IQR)):<br>Cases 31(28 to 35)<br>Controls (mean): 30.9(NA)<br>Nulliparous:<br>Cases 45%<br>Controls 45%<br>Pre-pregnancy BMI (median (IQR)):<br>Cases 32.4(27.6 to 36.2)<br>Controls 24.3(NA)
Walter et al. 2021 [48]<br>Retrospective cohort study<br>Brazil	n=132<br>Aim: Identify relationship between adherence to GWG recommendations and surgery-to-conception intervals.<br>Inclusion: Women aged ≥18, with singleton pregnancies post-RYGB, attending follow up appointments.	All RYGB.<br>Surgery-to-conception (median (IQR)): 30(16 to 49)	Maternal age: 33.3(4.7)<br>Multiparous: 39.3%<br>Pre-pregnancy BMI: 29(5.4)
Studies combining data from recipients of restrictive and malabsorptive bariatric surgery procedures			
Akhter et al. 2021[b] [26]<br>Case-control study<br>Belgium	n=122<br>Aim: Examine factors associated with SGA birth in women post-bariatric surgery.<br>Cases (n=25): Women with SGA offspring post-bariatric surgery.<br>Controls (n=97): Women with appropriate birthweight offspring post-bariatric surgery.	Cases<br>AGB 12%<br>SG 8%<br>RYGB 80%<br>BPD 0%<br>Controls<br>AGB 16.5%<br>SG 7.2%<br>RYGB 71.1%	Maternal age:<br>Cases 30.4(4.7)<br>Controls 30(4.5)<br>Nulliparous:<br>Cases 32%<br>Controls 30.9%<br>Pre-pregnancy BMI: Not reported

		BPD 3.1%<br>Surgery-to-conception:<br>Cases 40.9<br>Controls 50.0	
Ceulemans et al. 2021[b] [27]<br>Prospective cohort study<br>Belgium	n=127<br>Aim: Examine adherence to GWG recommendations in pregnant women post-bariatric surgery.<br>Inclusion: Women aged 18 to 45, with email access undergoing bariatric surgery at a single centre.	AGB 17%<br>GS 7%<br>RYGB 74%<br>BPD 2%<br>Surgery-to-conception:<br>Inadequate GWG 47(40)<br>Adequate GWG 47(35)<br>Excessive GWG 57(41)	Maternal age:<br>Inadequate GWG 29(4)<br>Adequate GWG 29(4)<br>Excessive GWG 30(4)<br>Nulliparous:<br>Inadequate GWG 33%<br>Adequate GWG 35%<br>Excessive GWG 32%<br>Pre-pregnancy BMI:<br>Inadequate GWG 28(7)<br>Adequate GWG 28(5)<br>Excessive GWG 29(5)
Coupaye et al. 2018 [29]<br>Retrospective cohort study<br>France	n=123<br>Aim: Assess the influence of serum micronutrients and GWG on foetal growth.<br>Inclusion: Singleton pregnancies of women post-SG or RYGB with at least one nutritional assessment.	SG 37%<br>RYGB 63%<br>Surgery-to-conception:<br>SG 24(18)<br>RYGB 31(22)	Maternal age:<br>SG 31.1(4.8)<br>RYGB 33.4(4.7)<br>Parity: Not reported<br>Pre-pregnancy BMI:<br>SG 31.6(6.8)<br>RYGB 31.2(5.0)
Ducrame et al. 2021 [33]<br>Prospective cohort study<br>France	n=87<br>Aim: Evaluate relationship between weight gain and micronutrient deficiency and pregnancy outcomes.<br>Inclusion: Pregnant women post-SG or RYBG with complete medical records.	SG 57%<br>RYGB 43%<br>Surgery-to-conception:<br>49.3	Maternal age: 32.4(5)<br>Nulliparous:<br>SG 27%<br>RYGB 32%<br>Pre-pregnancy BMI: 31.6(6.6)

Garretto et al. 2019 [34]<br>Retrospective cohort study<br>USA	n=119<br>Aim: Compare vitamin A consumption and rates of deficiency to controls.<br>Cases (n=53): Women with singleton pregnancy post-RYGB or SG, planning to breastfeed.<br>Controls (n=66): Women with a singleton pregnancy, without any malabsorptive surgery who are planning to breastfeed.	SG 54%<br>RYGB 46%<br>Surgery-to-conception:<br><12 months 26%	Maternal age:<br>Cases 31(5.5)<br>Controls 30(5.6)<br>Multiparous:<br>Cases 72%<br>Controls 68%<br>Pre-pregnancy BMI:<br>Cases 34(6.5)<br>Controls 30(7)
Getahun et al. 2022 [36]<br>Retrospective cohort study<br>USA	n= 20213<br>Aim: Compare GWG and pregnancy outcomes in women post-bariatric surgery to controls.<br>Cases(n=1886): Women who gave birth post bariatric surgery.<br>Controls (n=18327): Women with BMI >35kg/m^2 who gave birth in the same period without bariatric surgery.	AGB 6%<br>GS 47%<br>RYGB 45%<br>Surgery-to-conception:<br><12 months 19.7%<br>12-17 months 14.3%<br>18-23 months 11.6%<br>≥24 months 54.4%	Maternal age:<br>Cases 33.1(5.3)<br>Controls 31.1(5.1)<br>Nulliparous:<br>Cases 35%<br>Controls 34%<br>Pre-pregnancy BMI:<br>Cases 35(6.8)<br>Controls 43.1(4.8)
Grandfils et al. 2019 [37]<br>Retrospective cohort study<br>France	N=337<br>Aim: Examine adherence to GWG recommendations and pregnancy outcomes.<br>Inclusion: Any pregnancy post-AGB, SG or RYGB.	AGB 45.7%<br>SG 14.2%<br>RYGB 40.1%<br>Surgery-to-conception (median(IQR)):<br>Inadequate GWG 24(10 to 50)	Maternal age:<br>Inadequate GWG 30.6(4.6)<br>Adequate GWG 30.8(5.8)<br>Excessive GWG 31(5.5)<br>Nulliparous<br>Inadequate GWG 31.4%<br>Adequate GWG 32.2%

		Adequate GWG 30 (17.5 to 52)<br>Excessive GWG 36(20 to 58)	Excessive GWG 31.8%<br>Pre-pregnancy BMI:<br>Inadequate GWG 35.1(9.0)<br>Adequate GWG 31.6(7.2)<br>Excessive GWG 32.7(6.1)
Heusschen et al. 2022 [38]<br>Retrospective cohort study<br>Netherlands	n=196<br>Aim: Evaluate pregnancy outcomes according to adherence to WG recommendations and surgery-to-conception interval.<br>Inclusion: Pregnant women post bariatric surgery birthing at single centre.	SG 24%<br>RYGB 68%<br>OAGB 8%<br>Surgery-to-conception:<br>Inadequate GWG 26.1(22.9)<br>Adequate GWG 30.9(20.3)<br>Excessive GWG 41.1(24.3)	Maternal age:<br>Inadequate GWG 30.3(4.5)<br>Adequate GWG 29.3(4.9)<br>Excessive GWG 30.2(4.2)<br>Nulliparous<br>Inadequate GWG 52%<br>Adequate GWG 40%<br>Excessive GWG 43%<br>Pre-pregnancy BMI:<br>Inadequate GWG 32.7(8.7)<br>Adequate GWG 29.6(4.9)<br>Excessive GWG 29.9(4.3)
Jans et al. 2014 [39]<br>Prospective cohort study<br>Belgium	N=81<br>Aim: Investigate the incidence if vitamin K deficiency and the impact of supplementation on serum vitamin K.<br>Cases (n=49): Women aged >18 post-bariatric surgery presenting to antenatal care <15 weeks.<br>Controls (n=27) Pregnant women aged >18 without bariatric surgery, presenting for antenatal care <15 weeks.	AGB 36%<br>RYGB 61%<br>BPD 2%<br>Surgery-to-conception:<br>38.8(27.8)	Maternal age:<br>Cases 29.9(4.7)<br>Controls 29(4.5)<br>Nulliparous:<br>Cases 39%<br>Controls Not reported<br>Pre-pregnancy BMI:<br>Cases 28.4(5.5)<br>Controls 26(6.4)
Johansson et al. 2015 [40]	N=2952	AGB 2%<br>RYGB 97%<br>Other (not specified) 1%	Maternal age:<br>Cases 31(5)<br>Controls 31(5)

Retrospective cohort study<br><br>Sweden	Aim: Compare GWG, risk of GDM and other adverse pregnancy outcomes compared with controls.<br><br>Cases (n=596): Women who gave birth after BS during recruitment period.<br><br>Controls (n=2356): Women who gave birth in same period matched for age, pre-surgery BMI, smoking status (including number of cigarettes smoked), education and year of birth.	Surgery-to-conception (median (IQR)) 21.6(16.8 to 30)	Nulliparous:<br>Cases 40%<br>Controls 38%<br><br>Pre-pregnancy BMI:<br>Cases: 30.3(4.9)<br>Controls: 41.8(4.8)
Malik et al. 2020 [43]<br><br>Retrospective case-control study<br><br>Singapore	N=55<br><br>Aims: Compare total weight gain and pregnancy outcomes with controls.<br><br>Cases (n=24): Asian women who underwent bariatric surgery during study period.<br><br>Controls (n=31): Asian females with BMI >32.5kg/m2 without surgery, matched for age, parity, pre-surgery BMI and year of delivery.	AGB 6%<br>SG 81%<br>RYGB 13%<br><br>Surgery-to-conception: 33.7(29.1)	Maternal age:<br>Cases 32.7(4.8)<br>Controls 31.4(5.01)<br><br>Parity:<br>Cases 1.9(1.9)<br>Controls 1.3(1.1)<br><br>Pre-pregnancy BMI:<br>Cases 32.8(4.4)<br>Controls 40.8(6.9)
Walędziak et al. 2021 [47]<br><br>Retrospective cohort study<br><br>Poland	n=71<br><br>Aim: Compare mean GWG in women post-bariatric surgery with and without pregnancy complications.<br><br>Inclusion: Birth of live neonate post-bariatric surgery.	AGB 9.85%<br>SG 67.6%<br>RYGB 21.12%<br>OAGB 1.4%<br><br>Surgery-to-conception (mean (IQR)): 18(9 to 30)	Maternal age (median (IQR)): 34(31 to 39)<br><br>Parity: Not reported<br><br>Pre-pregnancy BMI (median (IQR)): 28.1(26.4 to 32.0)

[a]Overlapping study population from a single centre in Brazil.
[b]Overlapping study population from a study in Belgium.

Abbreviations: BMI = Body Mass Index, SD= Standard Deviation, IQR = Interquartile Range, AGB = Adjustable Gastric Band, SG = Sleeve gastrectomy, RYGB = Roux-en-Y Gastric Bypass, OAGB = One Anastomosis Gastric Bypass, BPD = Biliopancreatic Diversion, IQR = Interquartile range, SD= Standard deviation.

Supplement 3: Studies investigating dietary intake and supplementation

Reference, sample size (n=)	Dietary intake method and findings	Supplement dose	Supplement adherence	Incidence of maternal deficiency, (diagnosis of deficiency)	Maternal and neonatal outcomes
Studies including recipients of malabsorptive bariatric surgery procedures (RYGB, OAGB, BPD)					
Chagas et al. 2016 [28]<br>n=30	Not assessed	Multivitamin containing:<br>Zinc: 15 mg<br>Vitamin A: 5000 IU retinol	Measure not described.<br>Included participants adhering to ≥80% prescribed supplementation.	Retinol (<1.05µmol/L)<br>1st trimester 63%<br>2nd trimester 63%<br>3rd trimester 66%<br>β-carotene (<0.74µmol/L)<br>1st trimester 80%<br>2nd trimester 67%<br>3rd trimester 73%<br>Zinc (<10.1µmol/L)<br>1st trimester 20%<br>2nd trimester 17%<br>3rd trimester 20%	Not assessed
Cruz et al. 2020 [30]<br>n=109 (30 cases)	Not assessed	Multivitamin containing:<br>Vitamin D: 1500-2000 IU<br>Calcium: 1200 mg	Measure not described.<br>Included participants adhering to ≥80% prescribed supplementation.	Vitamin D (threshold unclear)<br>Controls:<br>≤12 months post-surgery 84%<br>>12 months post-surgery 91.1%<br>Cases in 1st trimester:<br>≤12 months post-surgery 60%<br>>12 months post-surgery 83.3%<br>Cases in the 2nd trimester:<br>≤12 months post-surgery 90%<br>>12 months post-surgery 86.7%<br>Cases in the 3rd trimester:<br>≤12 months post-surgery 80%	Pregnancy-induced hypertension (cases only)<br>≤12 months post-surgery 0%<br>>12 months post-surgery 3.3%<br>SGA (cases only)<br>≤12 months post-surgery 11.1%<br>>12 months post-surgery 0%<br>p0.03<br>LGA (cases only)<br>≤12 months post-surgery 11.1%<br>>12 months post-surgery 0%

				>12 months post-surgery 90%	p0.03
Cruz et al. 2017 [31]<br><br>n=30	Not assessed	Vitamin A: 500 IU retinol acetate<br>Iron: 90 mg	Measure not described.<br>Included participants adhering to ≥80% prescribed supplementation.	Anaemia (Hb <110g/L[a])<br>1st trimester 46.7%<br>2nd trimester 30%<br>3rd trimester 55.2%<br>Retinol (<1.05µmol/L)<br>1st trimester 63.3%<br>2nd trimester 63.3%<br>3rd trimester 65.5%<br>β-carotene (<0.74µmol/L)<br>1st trimester 80.0%<br>2nd trimester 66.7%<br>3rd trimester 75.9%	Not assessed
da Cruz et al. 2018 [32]<br>n=116 (39 cases)	Not assessed	Vitamin A: 5000 IU retinol acetate	Measured by counting number of pills taken.<br><br>Included participants adhering to ≥80% prescribed supplementation.	Iron deficiency anaemia (Hb <110g/L)<br>≤12 months post-surgery 71.4%<br>12 to 24 months post-surgery 75.0%<br>Retinol (<1.05µmol/L)<br>≤12 months post-surgery 57.1%<br>12 to 24 months post-surgery 78.1%<br>β-carotene (<0.74µmol/L)<br>≤12 months post-surgery 66.7%<br>12 to 24 months post-surgery 90.6%	Preterm birth (cases only)<br>≤12 months post-surgery 4.3%<br>12 to 24 months post-surgery 0%<br>No difference in mean retinol or β-carotene levels.<br>SGA (cases only)<br>≤12 months post-surgery 14.3%<br>12 to 24 months post-surgery 15.6%<br>No difference in mean retinol or β-carotene levels.
Medeiros et al. 2016 [44]<br>n=46	Not assessed	Calcium: 850 mg calcium carbonate	Measured by counting number of pills taken.	Vitamin D (<50 nmol/L[a])<br>1st trimester 30.4%<br>2nd trimester 19.6%<br>3rd trimester 39.1%	Pregnancy-induced hypertension<br>n=1

		Vitamin D: 600 IU	Included participants adhering to ≥80% prescribed supplementation.	Low serum calcium (<1.12 mmol/L)<br>1st trimester 15.2%<br>2nd trimester 15.2%<br>3rd trimester 20%	
Studies combining data from recipients of restrictive and malabsorptive bariatric surgery procedures					
Coupaye et al. 2018 [29]<br><br>n=123	4-day food diary obtained during pregnancy.<br><br>Energy (Kcal)<br>SG 1222 (425)<br>RYGB 1385(400)<br><br>Protein (g)<br>SG 46.7(14.5)<br>RYBG 59.1(20.9)<br><br>Carbohydrate (g)<br>SG 152(59)<br>RYGB 164(49)<br><br>Fat (g)<br>SG 47.3(20.3)<br>RYGB 54.8(19.1)	Elevit B9 (France):<br>Vitamin A 4000 IU retinyl palmitate<br>Folic acid 800 ug<br>Vitamin B12 4000 ug<br>Vitamin E 15 mg<br>Calcium 125 mg<br>Iron 60 mg<br>Copper 1 mg	Measure not described.<br><br>Multivitamin adherence:<br>SG 80%<br>RYGB 95%	Micronutrients measured in 2nd trimester<br>Low Hb (<97g/L[a])<br>SG 3%<br>RYGB 5%<br><br>Iron (iron <7.9 µmol/L)<br>SG 27%<br>RYGB 15%<br><br>Low ferritin (<2 µg/L)<br>SG 0%<br>RYGB 0%<br><br>Vitamin B12 (<130 ng/L)<br>SG 0%<br>RYGB 4%<br><br>Folate (<0.8 µg/L)<br>SG 0%<br>RYGB 2%<br><br>Vitamin D (<25 nmol/L)<br>SG 7%<br>RYGB 7%<br><br>Vitamin A (<1.22 µmol/L)<br>SG 30%<br>RYGB 31%	Birth weight z-score<br>Mean(SD) iron intake 90(6.8) mg from diet and supplements not a predictor.

				Vitamin E (<23 µmol/L)<br>SG 0%<br>RGYB 9%<br><br>Zinc (<7.8 µmol/L)<br>SG 0%<br>RYGB 0%	
Garretto et al. 2019 [34]<br><br>n=119 (n=53 cases)	Food frequency questionnaire at birth.<br><br>Vitamin A (µg)<br>Cases: 586(445)<br>Controls: 578(476)<br><br>β-carotene (µg)<br>Cases: 4925(4547)<br>Controls: 4134(3197)<br><br>α-carotene (µg)<br>Cases: 607(870)<br>Controls: 425(438)	Not reported.	Measure not described.<br><br>Adherence not reported, however analysis reports no difference in supplementation between groups.	Retinol (<1.05 µmol/L) in 3rd trimester<br>Cases 58%<br>Controls 62%	Preterm birth<br>Cases 19%<br>Controls 19%<br><br>Low birth weight<br>Cases 26%<br>Controls 12%<br><br>Macrosomia (>4000 g)<br>Cases 9%<br>Controls 17%
Jans et al. 2014 [39]<br><br>N=76 (n=49 cases)	7-day food record taken – data not reported.	Standard multivitamin supplement recommended, composition not reported.	Reports refusal to take vitamin K supplement, measure not described.<br><br>Supplement refusal:<br>1st trimester 90%<br>3rd trimester 84%	Vitamin K1 (<0.8 nmol/L, cases only)<br>1st trimester 80%<br>2nd trimester 59%<br>3rd trimester 50%	GDM (cases only)<br>AGB 7.7%<br>RYGB/BPD 5.6%<br><br>Pregnancy-induced hypertension (Cases only)<br>AGB 11.1%<br>RYGB/BPD 9.7%<br><br>Pre-eclampsia (Cases only)<br>AGB 0%<br>RYGB/BPD 3.2%

					Preterm birth (Cases only)<br>AGB 5.6%<br>RYGB/BPD 9.7%<br><br>Macrosomia (Cases only)<br>AGB 16.7%<br>RYGB/BPD 3.2%

[a] Units converted for consistency.

Abbreviations: SG = Sleeve gastrectomy, RYGB = Roux-en-Y gastric bypass, OAGB = One anastomosis gastric bypass, BPD = Biliopancreatic diversion, SGA = Small for gestational age, LGA = Large for gestational age.
